# Exploring glia to better understand Alzheimer’s disease

**DOI:** 10.1080/19768354.2018.1508498

**Published:** 2018-08-12

**Authors:** Yoo Sung Kim, Hae Myeong Jung, Bo-Eun Yoon

**Affiliations:** Department of Molecular Biology, Dankook University, Cheonan, Korea

**Keywords:** Alzheimer’s disease, microglia, astrocytes, neuroglia

## Abstract

The amyloid-β (Aβ) hypothesis has been the leading explanation for the pathogenesis of Alzheimer’s disease (AD). The most common traits of AD are cognitive impairments and memory loss, which are associated with the accumulation of Aβ. Aβ aggregates activate glial cells, which in turn remove Aβ. Because microglia act as immune cells in the brain, most glia-related studies of AD have focused primarily on this cell type. However, astrocytes, another type of glial cell, also participate in the brain immune system, synaptic formation, brain homeostasis, and various other brain functions. Accordingly, many studies on the underlying mechanisms of AD have investigated not only neurons but also glial cells. Although these studies suggest that microglia and astrocytes are effective targets for AD therapeutics, other recent studies have raised questions regarding whether microglial cells and/or astrocytes serve a neuroprotective or neurotoxic function in AD. To gain a better understanding of the mechanisms of AD and identify novel targets for AD treatment, in this review, we consider the role of both microglia and astrocytes in AD.

## Introduction

Alzheimer’s disease (AD) is a prototypical neurodegenerative disorder that usually manifests in late middle or old age. In 2016, about 47 million people were reported to suffer from dementia, a tremendous number that is predicted to increase to more than 131 million by 2050 (Prince et al. 2016). AD is the only disease among the top 10 potentially fatal diseases for which no treatments are available. Common symptoms of AD include dementia, progressive memory loss, dysfunctional thoughts, confusion, and changes in both traits and moods. It has long been thought that neuronal accumulation of amyloid-β (Aβ), a phenotype found in AD patients, is the main cause of AD (Bahmanyar et al. 1987). Although the primacy of Aβ in the etiology of AD has recently come into question, there is still a general consensus that Aβ is closely related to AD. Typically, this usually means that AD is studied in the context of Aβ and neurons. However, recent research has also considered AD in the context of glial cells, reflecting the growing interest in the pathophysiological role of these cells in the brain (Cairns et al. 1997). Most such studies have focused on microglia, the primary immune glial cell type in the brain. However, astrocytes—another glial cell type—are also involved in the immune system of the brain and in maintaining brain homeostasis. Accordingly, extending studies of AD to include a consideration of both microglia and astrocytes opens new avenues for identifying novel targets for AD treatment. Here, we consider AD in relation to microglia and astrocytes, and suggest future directions for AD-related research on glial cells.

## Results

Aβ is formed through a two-step proteolytic process in which amyloid precursor protein (APP) is cleaved by BACE1 (β-site APP-cleaving enzyme 1) to yield C99, which is further cleaved by γ-secretase to yield Aβ. The resulting Aβ monomers oligomerize with each other to form aggregates that are toxic to the brain and are postulated to cause AD (Wilquet and De Strooper 2004). Microglia, the primary immune cell type in the central nervous system (CNS), are closely associated with aging, especially during the period of AD development (Overmyer et al. 1999). Numerous studies have focused on the role of microglia in AD. However, because astrocytes function in synapse formation and neurotransmitter and ion homeostasis, and are linked to the brain immune system, this glial cell type has also come into focus in the context of AD research, especially given the close association between AD and cognitive impairments (Dossi et al. 2018). However, there is currently a debate regarding whether glial cells serve a neuroprotective or neurotoxic function in the brain. Here, we consider mainly this issue, addressing the function of both microglia and astrocytes.

## 1. Microglia as a neuroprotective cell in AD

The phagocytic function of microglia is activated in the brain in response to brain damage and diseases (Block et al. 2007). Synaptic and neuronal damage can be a major reason for dementia, especially in AD. Microglia serve a synaptic remodeling function that is suspected to eliminate Aβ plaques in the AD brain (Dickson et al. 1993). The role of microglia in the accumulation of Aβ was tested using microglia-ablated mice and control APP/PS1 mice, the latter of which harbor double mutations in APP and presenilin (PS) and exhibit detectable plaque deposition after 2 months of age (Grathwohl et al. 2009). Interestingly, no difference in Aβ accumulation was observed between the two genotypes. Although there is no evidence that microglia are involved in the formation of Aβ, clusters of receptors on microglia are responsible for both phagocytosis of fibrillar forms of Aβ and pinocytosis of soluble forms (Mandrekar et al. 2009). Because soluble Aβ moves through attachment to lipoproteins, the amount of Aβ in tissue is ∼50 times larger than that in cerebrospinal fluid (CSF). The movement of soluble Aβ by lipoprotein and degradation by microglia suggest potential new approaches for treating AD.

The idea that microglia could be neuroprotective in AD by degrading Aβ prompted the study of inflammation-related genes. An *in vitro* study of gray and white matter from AD patients’ brains showed an increase in interleukin-1β (IL-1β), IL-6, IL-8, tumor necrosis factor-α (TNF-α), macrophage chemotactic protein-1 (MCP-1), macrophage inflammatory protein-1α (MIP-1α), and macrophage colony stimulating factor (M-CSF) (Wang et al. 2015). However, in APP23-Transgenic mice which express human APP with Thy-1 promoter, repetitive inflammatory stimulation was shown to decrease the formation of Aβ plaques (Wendeln et al. 2018). The existence of TGF-β1, M-CSF, IL-34 or CX3CR1 (CX3C chemokine receptor 1) has been shown to facilitate the reduction in Aβ levels by microglia (Wyss-Coray et al. 2001; Mizuno et al. 2011). CX3CR1^CreER^ mice are also widely used to study AD and the influence of microglia; this is because CX3CR1, which, in the brain, is only expressed on microglia, is the only receptor for CX3CL1. Because microglia use chemokines as microenvironmental cues, CX3CR1 enables microglia to communicate with neurons. Using CX3CR1^CreER^ mice, Gan and colleagues (Parkhurst et al. 2013) showed that microglia regulate learning-dependent synapse formation in response to brain-derived neurotrophic factor (BDNF). Other receptors on microglia also help activate microglia. For instance, toll-like receptor (TLR)-2 and -4 activate microglia, promoting uptake of aggregated Aβ peptides (Jana et al. 2008; Song et al. 2011).

Microglia express neuroprotective traits by releasing moderate level of chemokines. These chemokines also help microglia to communicate with neurons. Aβ peptides are as neurotoxic, causing inflammation. These inflammatory responses by, i.e. IL-6, TNF-α activate microglia to the moderate extent. These early activation of microglia to Aβ helps microglia act on neuroprotective roles in AD.

## 2. Microglia as a neuroinflammatory cell in AD

Considering that microglia are able to interact with neurons and degrade Aβ in the brain, some studies have suggested that microglia serve a neuroprotective role in AD. Conversely, other reports have indicated that microglia are changed according to the phase of AD, reflecting neuroinflammation caused by Aβ, debris from deceased neurons, and chronic changes in the microenvironment. Even though microglia are newly recruited to senile plaques, microglia in old mice lose the ability to recognize and degrade Aβ. They are, however, consistently activated by Aβ plaques throughout the period of AD development and progression. Cytokines released by activated microglia, such as TNF-α, overactivate microglia. Overactivated microglia, in turn, are either suppressed through apoptosis or continue to activate other microglial cells (Block et al. 2007; Hickman et al. 2008). Microglia are commonly found near amyloid plaques in AD patients. However, such Aβ-surrounding microglia are not activated by adjacent Aβ; instead, microglia are recruited to the Aβ plaque from plaque-free areas (Füger et al. 2017). Microglia are better able to move from plaque-free areas to Aβ plaque-adjacent regions in the AD brain than in the normal adult brain (Eyo et al. 2018). Aβ itself is also neurotoxic and decreases the viability of neurons or promotes their degeneration (Rogove and Tsirka 1998). Consequently, microglia are less able to degrade Aβ, and Aβ accumulates (Wang et al. 2015). Because microglia are overactivated by released cytokines and chronic neuronal damage, inflammation and immune responses cause microglia to release excessive cytokines. Consistent with this, AD patients with slight dementia show a greater increase in the level of cytokines than patients without dementia. The excitatory neurotransmitter, glutamate, is released into the synaptic cleft and increases the level of intracellular Ca^2+^, which in turn activates neurons and glial cells, causing more release of glutamate (Hynd et al. 2004). The neuronal death caused by excess glutamate is blocked by removing Ca^2+^ or blocking the *N*-methyl-d-aspartate receptor (NMDAR), an ionotropic glutamate receptor (Choi et al. 1987; Moriguchi et al. 2003). This neurotoxicity has motivated researchers to seek alternative AD treatments.

Summarizing the functions of microglia, even with the neuroprotective functions we have read above, overactivation of microglia also acts the neurotoxic role in the brain. In chronic AD condition, microglia induce neuronal death by excessive amount of glutamate release and surrounding microglial cell death. Decreased number of microglia affects to the reduction of Aβ plaques constituting the toxic role of microglia.

## 3. Astrocytes as a neuroprotective cell in AD

Because glial cells interact with each other, it is not surprising that microglia also associate with astrocytes. Astrocytes are activated by nearby Aβ (Kim and Yoon 2017), causing an increase in intracellular Ca^2+^ concentration and uptake of Aβ (Tyurikova et al. 2016). Astrocytes are also activated by cytokines and chemokines released from microglia, such as IL-1, TNF-α, and TGF-β (Neumann et al. 2008). These activated astrocytes release numerous immune system-related factors, such as TNF-α, TGF-β, IL-1β, and IL-6. To date, neurons have been the main focus of studies investigating AD treatment strategies; however, astrocytes have recently come to the fore as another target for AD therapeutics. Given the heterogeneity of the brain microenvironment, a dual focus on astrocytes and neurons makes biological sense. As noted above, ApoE2 proteins, which help to degrade the soluble form of Aβ, are primarily produced and secreted by astrocytes. ATP-binding cassette transporter (ABCA1) is closely associated with ApoE lipidation. Interesting in this context, it has been reported that astrocyte-conditioned medium from *Abca1*-knockout mice is unable to degrade Aβ, whereas medium conditioned by astrocytes from WT mice retains this ability (Jiang et al. 2008). Moreover, astrocytes in cultures of brain tissue are only activated in the presence of Aβ. An *in vitro* study investigating the response of adult astrocytes cultured on Aβ-coated plates revealed that even adult astrocytes are capable of degrading extracellular Aβ (Wyss-Coray et al. 2003). The beneficial effects of activated astrocytes are limited, however, as an excessive number of such astrocytes is thought to be detrimental.

Briefly describing the neuroprotective role of astrocytes, early release of inflammatory cytokines from microglia activates astrocytes. Activated astrocytes not only activate nearby microglial cells, i.e. TNF-α, TGF-β, IL-1β, but degrade soluble form of Aβ by apolipoproteins, especially ApoE2. These astrocytes are thought to be useful as therapeutic target for AD, because astrocytes acquire vast portion in the brain.

## 4. Astrocytes as a neurotoxic cell in AD

Although some studies have touted the neuroprotective function of astrocytes, others have suggested that reactive astrocytes are neurotoxic from the outset. Astrocytes in the AD brain have lost the ability to maintain their ionic balance, particularly with respect to Ca^2+^. In this AD setting, Aβ activates astrocytes, inducing an increase in intracellular Ca^2+^ that stimulates NADPH oxidase (NOX), causing neuronal death through oxidative stress; inducible nitric oxide synthase (iNOS) is also upregulated in these activated astrocytes (Abramov and Duchen 2005). Consistent with this, the L-type Ca^2+^ channel blockers nimodipine and verapamil exert a protective effect on neurons by inhibiting excessively activated microglia and astrocytes (Hashioka et al. 2012). Other *in vitro* studies using cultured astrocytes have demonstrated interactions between microglia and astrocyte, showing that microglia do not phagocytose senile plaques when co-cultured with astrocytes, but quickly do so when cultured alone (DeWitt et al. 1998). In contrast, another study demonstrated synergetic functions of astrocytes and microglia, reporting that stromal cell-derived factor-1α (SDF-1α) activates astrocytes, which subsequently activate microglia. These activated microglia secrete TNF-α, which feeds back on astrocytes to stimulate the release of glutamate. As noted above, excessive amounts of released glutamate are excitotoxic (Bezzi et al. 2001). Unlike the case with cytokines and chemokines, apolipoprotein E is present in different genetically important isoforms, including ApoE2, ApoE3, and ApoE4. ApoE3 and ApoE4 are both neurotoxic, but ApoE4 is more harmful because it serves to support Aβ deposition from the initial stages of AD and increases Aβ residence time (Liu et al. 2017). Some other studies have investigated the possible neurotoxicity of astrocytes by blocking intracellular signaling pathways in astrocytes. In one such study using APP/PS1 mice, the inhibition of the calcineurin pathway was shown to decrease the number of reactive astrocytes, resulting in a reduction in Aβ concentration and improved cognitive function. In another possible connection between reactive astrocytes and AD, it has been shown that the activation of astrocytes by Aβ induces the synthesis of GABA by monoamine oxidase B (MAOB), resulting in an increase in the tonic release of GABA from astrocytes (Jo et al. 2014). These astrocytic tonic GABA nowadays take spotlight for the importance as the regulator of neuronal activity and physiological functions (Woo et al. 2018) which can make sense to astrocytes affecting pathological state (Kim et al. 2017).

Similar with microglia, overactivated astrocytes induce neurotoxic function, with excessive amount of glutamate, and interactions with microglia. Suppressing the phagocytosis of microglia by existing with astrocytes. Another essential gene from the neuroprotective role of astrocyte, ApoE, became neurotoxic by having isoform of ApoE3 or ApoE4. Both microglia and astrocytes are doing neuroprotective role in the early stage of AD, but with chronic existence of Aβ, they serve neurotoxic role and aggravate pathological states.

## Conclusion

The Aβ hypothesis has long been at the forefront of the debate surrounding the mechanism of AD induction. The development of this hypothesis reflects the results of studies ranging from the investigation of processing events leading to Aβ protein production, to oligomerization and deposition, and ultimately degradation. Given the great threat posed by AD, considerable research effort has been devoted to developing therapeutic strategies for treating AD. Since the first description of AD in 1906, most research has focused on the only expected target—the neuron. However, with little mechanistic insight into the pathogenesis of AD, therapeutic strategies remained elusive. Starting about 15 years ago, researchers began to approach AD from a different perspective. The focus of this new approach has been on microglia, a prominent immunological cell in the brain, and astrocytes, the most abundant glial cell type. It stands to reason that glial cells could be a powerful therapeutic target, given recent insights into glial cell and neuron functions, importantly including a better understanding of neuron–glia interactions.

**Figure 1.**
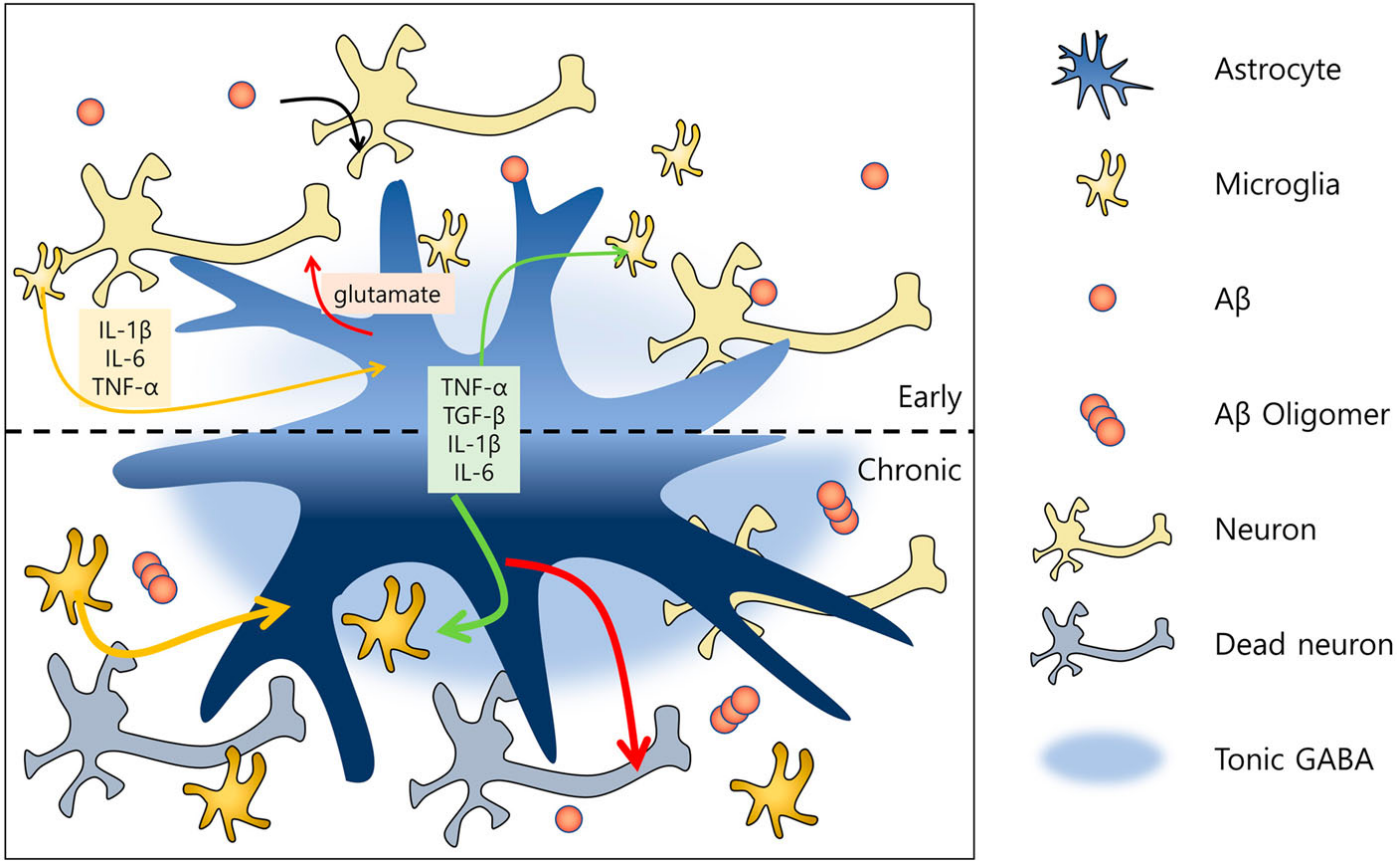
Representative schematic of function of glial cells during the progression of AD. In the early stage of Alzheimer’s disease, microglial cells are activated by amyloid beta proteins and release cytokines and chemokines (e.g. IL-1β, IL-6, TNF-α). Reactive microglia then activate astrocytes, which release TNF-α, TGF-β, IL-1β and IL-6, as well as certain neurotransmitters (e.g. glutamate) that reciprocally influence microglia. The communication of glial cells and neurons with Aβ induces a chronic disease stage. In this phase, microglia and astrocytes positively feedback on each other, and are thus continuously activated. Excessive amounts of glutamate then become sufficiently neurotoxic to kill the neurons. At this neuronal death stage, increased tonic release of GABA by reactive astrocytes is detected.

In this review, we have considered possible roles of microglia and astrocytes in AD from both protective and toxic perspectives. Briefly summarized, as AD progresses, microglia react to Aβ plaques, becoming activated. Cytokines and chemokines released from microglia activate astrocytes, which in turn also release cytokines into the microenvironment. Cytokines released from microglia and astrocytes feedback on microglia and astrocytes to stimulate their activation. This positively reinforcing cycle creates a chronic inflammatory stimulus for glial cells; as the disease progresses, microglia lose their protective function and become neurotoxic. Increased TNF-α and increased glutamate cause neuronal excitotoxicity, an effect that is exacerbated by increased tonic release of GABA from activated astrocytes (Figure 1).

Despite controversies surrounding the effects of microglia and astrocytes in AD, these cell types nonetheless represent another avenue for treating AD. If reactive microglia and astrocytes are neuroprotective, the prediction is that the co-existence of reactive microglia and astrocytes with Aβ reflects a response to the deposition of Aβ plaques, suggesting that therapies designed to enhance microglial and astrocyte reactivity might be effective. Conversely, if reactive microglia and astrocytes are neurotoxic and induce the formation of Aβ oligomers, it would present an opportunity to identify strategies for preventing reactivation of microglia and inhibiting astrogliosis.
